# Protective effects of genetic inhibition of Discoidin Domain Receptor 1 in experimental renal disease

**DOI:** 10.1038/srep21262

**Published:** 2016-02-16

**Authors:** Monique Kerroch, Carlo Alfieri, Aude Dorison, Jean-Jacques Boffa, Christos Chatziantoniou, Jean-Claude Dussaule

**Affiliations:** 1 INSERM UMR S 1155, Hôpital Tenon, 75020 Paris, France; 2 Sorbonne Universités, UPMC Univ Paris 06, Paris, France; 3 Department of Medicine and Medical Specialties, Unit of Nephrology, Dialysis, and Renal Transplant, Fondazione Istituto di Ricerca e Cura a Carattere Scientifico Ca‘ Granda Ospedale Maggiore Policlinico, Milan, Italy; 4 Service de Néphrologie et Dialyses, Hôpital Tenon, AP-HP, Paris, France; 5 Department of Physiology, Saint-Antoine Hospital, AP-HP, Paris, France

## Abstract

Chronic kidney disease is a progressive incurable pathology affecting millions of people. Intensive investigations aim to identify targets for therapy. We have previously demonstrated that abnormal expression of the Discoidin Domain Receptor 1 (DDR1) is a key factor of renal disease by promoting inflammation and fibrosis. The present study investigates whether blocking the expression of DDR1 after the initiation of renal disease can delay or arrest the progression of this pathology. Severe renal disease was induced by either injecting nephrotoxic serum (NTS) or performing unilateral ureteral obstruction in mice, and the expression of DDR1 was inhibited by administering antisense oligodeoxynucleotides either at 4 or 8 days after NTS (corresponding to early or more established phases of disease, respectively), or at day 2 after ligation. DDR1 antisense administration at day 4 stopped the increase of proteinuria and protected animals against the progression of glomeruloneprhitis, as evidenced by functional, structural and cellular indexes. Antisense administration at day 8 delayed progression –but to a smaller degree- of renal disease. Similar beneficial effects on renal structure and inflammation were observed with the antisense administration of DDR1 after ureteral ligation. Thus, targeting DDR1 can be a promising strategy in the treatment of chronic kidney disease.

Renal fibrosis is the consequence of the accumulation of extracellular matrix in the renal parenchyma. This pathological process affects glomerular filtration rate and may lead to dialysis or kidney transplantation. Regardless of the origin of renal injury, all the compartments are implied in the degradation of renal function, which suggests the involvement of common mechanisms in the progression of renal disease.

Discoidin Domain Receptor 1 (DDR1) is present in a variety of tissues such as brain, lung, kidney, spleen, and placenta, predominantly in epithelial cells. DDR1 are also expressed in the nervous system and in cells of the immune system, but a systematic analysis of the precise cellular distribution of DDR1 in different tissues has not yet been performed. DDR1 specifically bind a number of different native collagen types. This binding leads to phosphorylation of tyrosine kinases located in the intracellular part of the receptor, dimerization and activation of signaling pathways involved to cell migration, adhesion and inflammation such as ERK1/2, p38, PI3K, Src, Stat1/3. Interestingly, DDR1 is the only known member of tyrosine kinase receptor family to be directly activated upon binding to components of the extracellular matrix. DDR1 overexpression and/or acivation has been reported in various pathologies such as fibrosis, inflammation, arthritis or cancer^1^,^2^. Deletion of the DDR1 gene prevented renal inflammation and fibrosis in three models of renal disease, hypertensive nephroangiosclerosis, unilateral ureteral obstruction (UUO) and nephrotoxic serum (NTS)-induced glomerulonephritis^3^,^4^,^5^. In the glomerular model of renal disease, we showed that preventive administration of oligodeoxynucleotides (ODNs) to inhibit DDR1 expression protected kidneys with an efficiency close to that of gene deletion.

In the previous studies mentioned above, we did not address the question of a therapeutic use of DDR1 inhibition. The aim of the present study was to investigate if in experimental models of renal disease such as the NTS-induced glomerulonephritis and the UUO, the inhibition of DDR1 expression obtained by injecting specific antisense ODNs after the initiation of renal injury would affect the final outcome of renal disease progression, validating thus the concept that DDR1 can be a target for therapy. Our second objective was to evaluate the kinetics of DDR1 involvement in the progression of renal failure in this model by studying the beneficial effects of ODNs administered at an early phase compared to a more advanced stage of the disease.

## Results

### DDR1 expression is inhibited by DDR1 antisense administration

**Figure 1:**
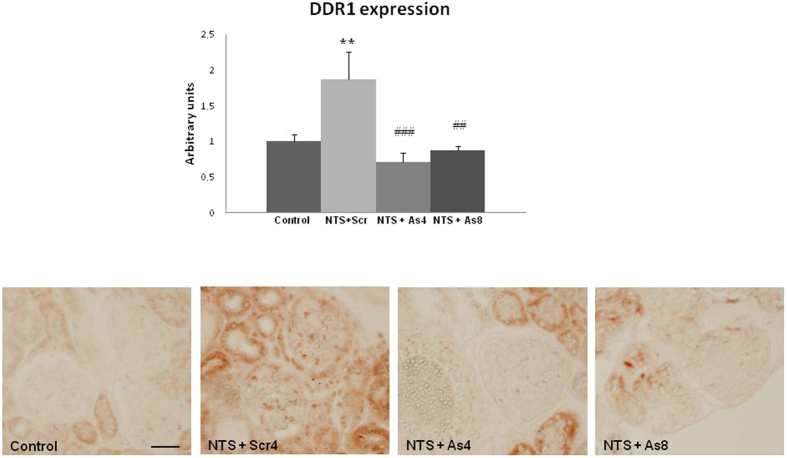
DDR1 antisense administration inhibited the NTS-induced increase of renal expression of DDR1. Upper: DDR1 mRNA expression at day 15 in control and NTS mice receiving DDR1 antisense treatment since 4^th^ (NTS + As4) or 8^th^ (NTS + As8) day. n = 5–10. **p < 0.01 vs Control; ^##^p < 0.01, and ^###^p < 0.001 vs NTS + Scrambled. Lower: Representative examples of DDR1 immunostaining at day 15 in the cortex of mice treated or not with DDR1 antisense from day 4 or 8 (scale bar = 20μm).

In agreement with previous studies^5^), DDR1 expression was induced in the renal cortex following NTS administration (Fig. 1, upper). In previous studies we have shown that this *de novo* expression of DDR1 was induced within glomeruli and particularly in podocytes, because it co-localized with nephrin^5^). DDR1 antisense treatment blocked the increase of DDR1 mRNA expression and inhibited the glomerular expression of DDR1 at day 15 (Fig. 1, lower panel).

### Inhibition of DDR1 arrested the progression of renal disease

**Figure 2:**
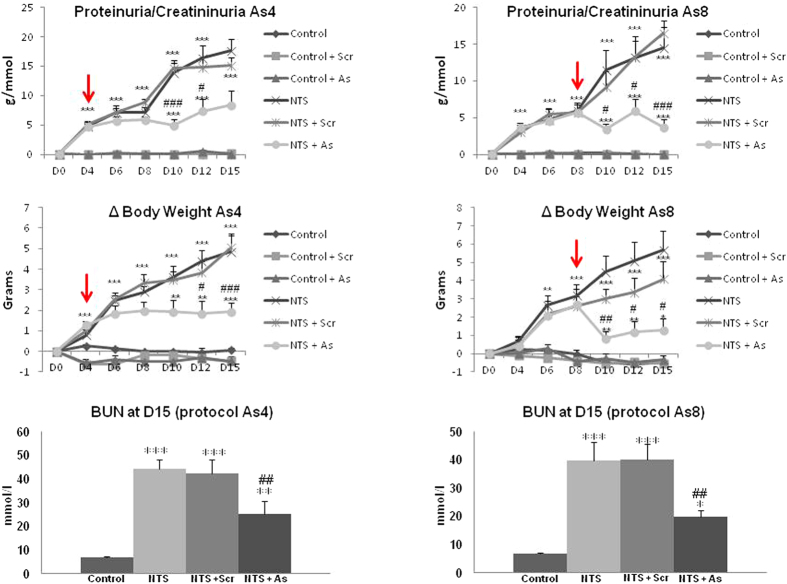
DDR1 antisense treatment stopped increases of proteinuria, body weight and blood urea nitrogen (BUN) that are induced by NTS administration. Red arrows indicate the beginning of ODNs treatment. n = 5–10. *p < 0.05, **p < 0.01 and ***p < 0.001 vs Control ; ^#^p < 0.05, ^##^p < 0.01 and ^###^p < 0.001 vs NTS + Scrambled.

As expected, NTS administration progressively degraded renal function as evidenced by the values of proteinuria, body weight increase and uremia (Fig. 2). Administration of DDR1 antisense at day 4- or 8- stabilized proteinuria, body weight intake and uremia (Fig. 2), thus indicating an arrest of the progression of renal disease. At the end of the treatment (day 15) renal function of animals treated with DDR1 antisense was clearly preserved compared to NTS− or NTS + scrambled treated animals in which renal disease continued to progress (Fig. 2).

### Administration of DDR1 antisense protected against the deterioration of renal structure

**Figure 3:**
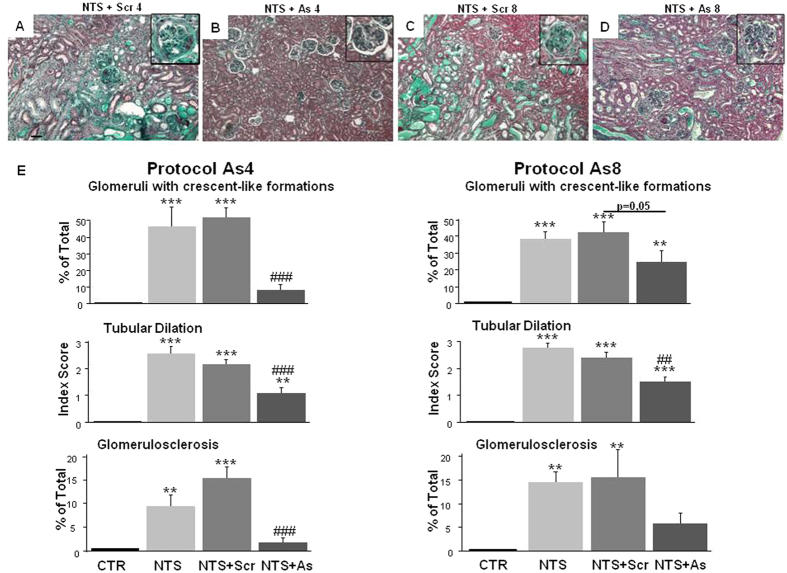
DDR1 antisense treatment alleviated renal histological damages induced by NTS administration. (**A**–**D**) Representative views of Masson’s trichrome coloration on renal sections at day 15 in NTS + Scr4, NTS + As4, NTS + Scr8 and NTS + As8 mice (scale bar = 50μm). (**E**) Estimation of glomeruli containing crescents (**upper**), tubular dilation (middle) and glomerulosclerosis (**lower** panels) at day 15. n = 10–15. **p < 0.01, and ***p < 0.001 vs Control; ^#^p < 0.05, ^##^p < 0.01, and ^###^p < 0.001 vs NTS + Scrambled.

NTS-induced renal disease was associated with severe histological alterations as 45% of glomeruli at day 15 presented crescent-like formations, tubular dilation increased to 2.5 (in a scale from 0–4), and 15% of glomeruli were sclerotic (Fig. 3). Administration of scrambled ODNs did not have any effect on these parameters renal structure and function. In contrast, administration of antisense ODNs targeting DDR1 at day 4 or 8 stopped the deterioration of the renal structure and resulted in a significant protection of renal tissues at day 15 (Fig. 3). The protection of the antisense treatment started at day 8 was less efficient compared to antisense treatment started at day 4 (Fig. 3).

**Figure 4:**
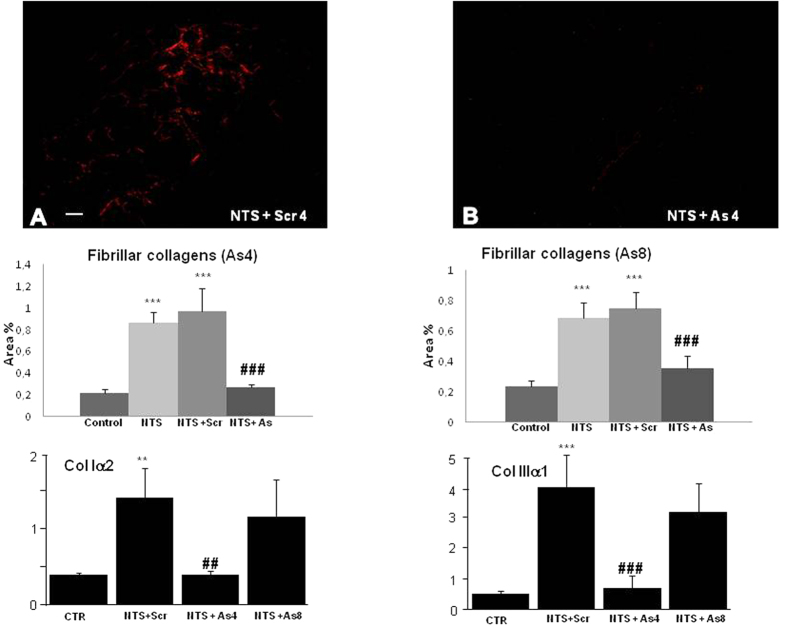
Accumulation of fibrillar interstitial collagen induced by NTS was arrested in mice treated with DDR1 antisense. Upper: Representative views (scale bar = 50μm) of Sirius Red coloration on renal sections at day 15 in NTS + Scr4 (**A**) and NTS + As4 (**B**) mice showing accumulation of interstitial fibrillar collagen. Middle: Quantification of fibrillar collagen at day 15 in As4 and As8 protocols. n = 10–15. ***p < 0.001 vs Control; ^###^p < 0.001 vs NTS + Scrambled. Lower: mRNA expressions of collagen I α2 and collagen III a1 in mice treated either with As or with Scr from day 4 or 8 and sacrificed at day 15. n = 4–10; **p < 0.01, and ***p < 0.001 vs Control; ^##^p < 0.01, and ^###^p < 0.001 vs NTS + Scrambled.

Fibrosis, evaluated by Sirius Red positive areas (Fig. 4) and collagen 1-α2 or collagen 3-α1 mRNA expressions increased 4–8 fold in NTS or NTS + scrambled mice (Fig. 4 middle and lower panels). DDR1 antisense administration at day 4 blocked the fibrotic response (middle and lower panels). When the antisense treatment started at day 8, the anti-fibrotic protection measured by Sirius red staining at day 15 was significant (Fig. 4, middle), but less efficient for collagen 1-α2 and collagen 3-α1 mRNA expressions (Fig. 4, lower).

### Inhibition of DDR1 stopped the renal inflammatory influx

**Figure 5:**
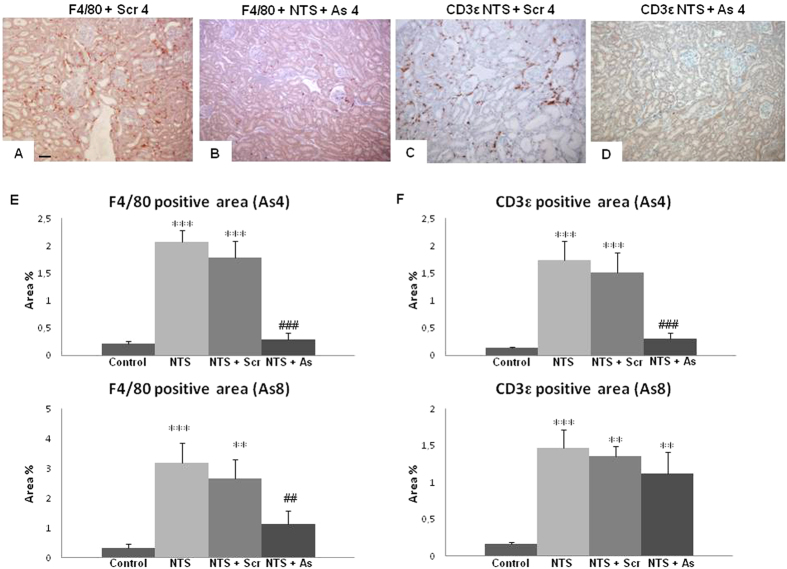
Infiltration of inflammatory cells was reduced in mice treated with DDR1 antisense. Representative examples of F4/80 (**A**,**B**) and CD3ε (**C**,**D**) immunostaining on renal sections in NTS + Scr4 and NTS + As4 mice showing the presence of macrophages and T-lymphocytes in renal parenchyma (scale bar = 50μm). (**E**) Evaluation of the percentage of positive area for F4/80 (**left**) and CD3ε (**right**) staining at day 15 in As4 and As8 protocols. n = 10–15. **p < 0.01, and ***p < 0.001 vs Control; ^##^p < 0.01, and ^###^p < 0.001 vs NTS + Scrambled.

Inflammatory infiltration was studied by immunostaining of macrophages and dendritic cells with a F4/80 antibody and of T lymphocytes with a CD3ε antibody. Again, starting antisense treatment at day 4 provided an almost complete protection against inflammatory influx at the end of the protocol (day 15, Fig. 5B,D,E). Starting antisense treatment at day 8 was less efficient: it provided a relative protection against macrophages at day 15 (Fig. 5E lower left), but had limited effect on lymphocyte infiltration (Fig. 5E lower right).

### DDR1 antisense treatment inhibited the synthesis of pro-fibrotic and pro-inflammatory mediators

**Figure 6:**
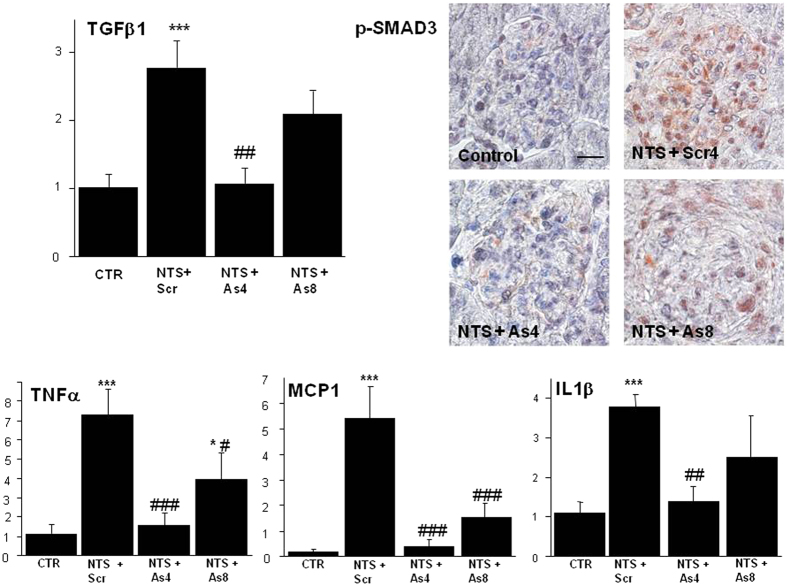
Treatment with DDR1 antisense inhibited the synthesis of pro-fibrotic and pro-inflammatory mediators. Upper left: Expression of TGFβ in mice treated either with As or with Scr from day 4 or 8 and sacrificed at day 15. n = 4–10; ***p <  0.001 vs Control; ^##^p < 0.01 vs NTS + Scrambled. **Upper right**: Representative examples of p-Smad3 staining at day 15 in the cortex of mice treated or not with DDR1 antisense from day 4 or 8 (scale bar = 10μm). Lower panels: Expressions of TNFα, MCP-1 and IL1β in mice treated with As or Scr from day 4 or 8 and sacrificed at day 15. n = 4–10; *p < 0.05, and ***p <  0.001 vs Control; ^#^p < 0.05, ^##^p < 0.01 and ^###^p < 0.001 vs NTS + Scrambled.

As shown in Fig 6 (upper left panel) DDR1 antisense administration blocked the NTS-induced increase of TGFβ1 expression. This inhibition affected the profibrotic pathway of TGFβ as evidenced by the negligible expression of p-Smad3 in the glomeruli of antisense-treated mice (Fig. 6, upper right panels). To confirm the protection induced by the blockade of DDR1 synthesis against inflammation we measured mRNA expression of several inflammatory mediators. As expected from previous studies, NTS administration increased mRNA expression of TNFα, MCP-1 and IL1-β, (Fig. 6 lower panels). Antisense treatment stopped the induction of TNFα after day 4 as well as its further activation after day 8 (Fig. 6, lower left panel). A similar observation is made with MCP-1 and IL1-β, (Fig. 6, lower panels).

### Administration of DDR1 antisense inhibited the UUO-induced increase of renal expression of DDR1

**Figure 7:**
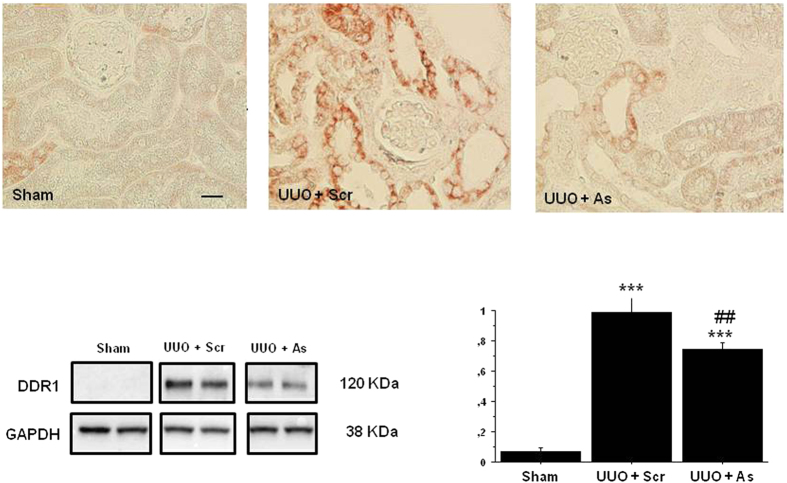
DDR1 antisense administration inhibited the UUO-induced increase of renal expression of DDR1. Upper: Representative examples of DDR1 immunostaining at day 7 in the cortex of mice treated or not with DDR1 antisense from day 2 (scale bar = 20μm). Lower left: Representative examples of Western blotting of DDR1 expression at day 7 in sham and UUO mice receiving DDR1 scrambled or antisense treatment since day 2. All gels have been run under the same experimental conditions. Lower right: Quantification of protein expression, n = 4–12. ***p < 0.001 vs Control; ^##^p < 0.01 vs UUO + Scrambled.

DDR1 expression was strongly induced in the damaged renal cortex following ureteral ligation (Fig. 7, upper panel). As was the case with the NTS model, DDR1 antisense treatment inhibited the tubulointerstitial expression of DDR1 at day 7. The overexpression of DDR1 and the efficiency of antisense treatment was confirmed by WB quantification of DDR1 protein expression (Fig. 7, lower panel).

### DDR1 antisense treatment alleviated renal histological damages and inflammation induced by UUO

**Figure 8:**
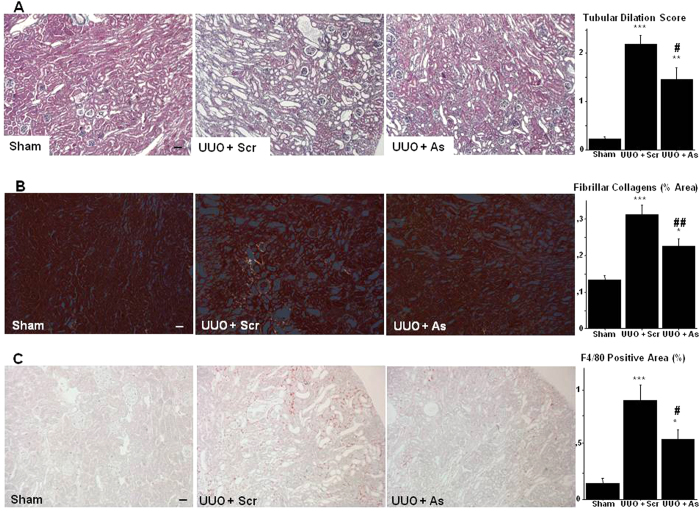
DDR1 antisense treatment alleviated renal histological damages and inflammation induced by UUO. (**A**) Representative views of Masson’s trichrome coloration on renal sections at day 7 in Sham, UUO + Scr and UUO + As mice (scale bar = 50μm). **Right**: Estimation of tubular dilation at day 7. n = 4–12. **p < 0.01, and ***p < 0.001 vs Control; ^#^p < 0.05, vs UUO + Scrambled. (**B**) Representative views of Sirius Red coloration on renal sections at day 7 in Sham, UUO + Scr and UUO + As mice showing accumulation of interstitial fibrillar collagen (scale bar = 50μm). **Right**: Quantification of fibrillar collagen. n = 4–12. *p < 0.05 and ***p < 0.001 vs Control; ^##^p < 0.01 vs UUO + Scrambled. (**C**) Representative examples of F4/80 immunostaining on renal sections in Sham, UUO + Scr and UUO + As mice showing the presence of macrophages in renal interstitium (scale bar = 50μm). **Right**: Evaluation of the percentage of positive area for F4/80 staining. n = 4–12. *p < 0.05, and ***p < 0.001 vs Control; ^#^p < 0.05 vs UUO + Scrambled.

Tubular dilation, renal fibrogenesis and inflammatory influx are typical major histological events following UUO. Compared to the kidneys receiving scrambled sequences, administration of antisense ODNs targeting DDR1 at day 2 after the ligation resulted in a significant protection of renal cortex as evidenced by the lesser degree of tubular dilation (Fig. 8A), the decreased formation of fibrillar collagens (Fig. 8B) and the reduced infiltration of monocytes/macrophages (Fig. 8C).

## Discussion

DDR1 is a tyrosine kinase transmembrane receptor of collagens and is expressed in several cell types and organs^1^,^2^,^6^,^7^,^8^. The interesting feature of DDR1 is that after the binding of collagens, this receptor is dimerized leading to phosphorylation of tyrosine-kinase and depending on the cellular context, can trigger various signaling pathways such as P38 kinase, MAP ER1/2 kinase, PI3 kinase or JNK pathways^6^,^7^. Since DDR1 has this dual function, a collage receptor which can activate inflammatory signaling pathways, we have investigated the involvement of DDR1 activation in mechanisms promoting renal fibrosis and inflammation.

In these previous works, we have demonstrated that mice lacking expression of DDR1 are preserved against the development of renal injuries in several models of kidney disease^3^,^4^,^5^. Thus, in a model of angiotensin II-induced hypertensive nephropathy, DDR1 null mice were protected against proteinuria, perivascular and periglomerular inflammation, glomerulosclerosis and interstitial fibrosis^3^. Data from studies in the UUO model indicated that DDR1 promotes renal disease through activation of the inflammatory response since macrophages from DDR1-deleted animals displayed impaired migration in response to MCP1^4^. Other investigators observed that in a model mimicking the Alport’s syndrome (COL4A3^−/−^ mice), deletion of DDR1 delays renal fibrosis via inhibition of NF-κB, IL-6 and TGF-β signaling^9^. Subsequent studies showed that in the NTS model, DDR1 expression is induced and progressively increased in podocytes. DDR1 null mice were protected against renal disease as evidenced by decreased proteinuria, glomerular inflammation and fibrosis, and increased survival^5^. Interestingly, it appears that DDR1 can be expressed and activated in infiltrating or resident cells, depending on the experimental model i.e. in macrophages and tubular epithelial cells in the UUO model^4^, in smooth muscle cells in hypertensive nephropathy^3^ and in podocytes in glomerulonephritis^5^. In the NTS model, a prevention was also observed in wild type mice treated with specific antisense ODNs blocking the expression of DDR1^5^. Use of a general knockout mice or administration of antisense before the induction of the disease are preventive approaches because the expression of DDR1 is either null or inhibited before the beginning of the disease. In pathologies like renal disease preventive treatments are not applicable, and although several experimental studies have shown that shutting down the expression of a gene before the initiation of the disease can prevent the decline of renal function, only in a few cases blockade of this gene after the initiation of the disease was efficient in protecting the kidney. For this reason, in the present study a major objective was to investigate the effects of the pharmaco-genetic inhibition of DDR1 as a therapy approach after the beginning of the disease. Such an approach has been successfully realized in the past, for instance with the delayed blockade of bradykinin receptors^10^, the inhibition of growth factors^11^ or the activation of Il-10^12^.

In the NTS protocol, the DDR1 antisense administration started either at day 4, or later at day 8. Classically investigators are considering days 2–4 as an ‘early’ phase of glomerulonephritis, and days 7–9 as more advanced phases. Based on our experience and previous studies, proteinuria and body weight intake are significantly increased at day 4 with no severe alterations of renal structure, whereas at day 8, in addition to proteinuria and body weight intake, several parameters of renal structure are altered such as appearance of tubular dilation and crescent-like formations. Thus, although the differences in renal function can appear minimal between day 4 and day 8, still important differences exist between these time points regarding the histological alterations. In both timings of treatment, DDR1 antisense administration stopped the progression of renal disease and the indexes of renal function did not decline furthermore. As result, at the end of the protocol at day 15, the groups of animals receiving antisense treatment were protected compared to those receiving NTS either alone or combined with scrambled ODNS in all measured functional or histological parameters. However, the antisense treatment was not able to induce a complete regression to all renal lesions. Proteinuria, uremia and renal histology were not normalized even when the treatment was started at day 4. This finding indicates that DDR1 most likely behaves as an essential amplifier of the fibrotic and inflammatory injury, but not as an initiator of these pathological processes^13^.

Ureteral obstruction is characterized by an immediate arrest of filtration followed by inflammatory response and activation of fibrogenesis. Based on our previous experience, the DDR1 antisense treatment started at day 2 corresponding to an early phase in which tubular dilation, inflammatory influx and abnormal deposition of extracellular matrix are clearly observed^14^. As was the case with the NTS model, antisense treatment efficiently decreased DDR1 expression and protected kidneys from the UUO-induced alterations of renal structure and inflammation.

We identified several actors of the inflammatory process to be associated with DDR1 expression such as MCP-1, IL1β, and TNFα^15^,^16^,^17^,^18^,^19^,^20^. As was the case with mice lacking DDR1 gene expression^3^,^4^,^5^, we observed that antisense treatment was associated with decreased inflammatory influx. This interaction between DDR1 expression and inflammation could be due either to an activation of DDR1 in inflammatory circulating cells and/or to the local tissue expression of DDR1 inducing cytokines and driving inflammation. In an *in vitro* setting, DDR1 was an important mediator in the migration of T cells through collagen^21^,^22^. However, the observed staining of DDR1 in the kidney following NTS administration or ureteral obstruction does not appear to be in T lymphocytes (Figs 1 and 7). Similarly, we did not observe DDR1 expression on T-lymphocytes in our previous studies with the other models of renal injury^3^,^4^,^5^. In addition, the data in Fig. 6 show that DDR1 activation was accompanied by increased expression of pro-inflammatory cytokines such as TNFα, MCP-1 and IL1β. The observation that the antisense-induced decrease of DDR1 expression reduced the expression of these cytokines suggest a link between DDR1 and these signaling pathways. A similar result was previously observed where *in vitro* transfection of podocytes with DDR1 was accompanied by the induction of pro-inflammatory cytokine IL1-β^5^. It appears thus that in response to an aggression, renal cells start expressing DDR1 which is activated by collagens to induce production of cytokines and amplify the detrimental interaction between renal inflammation-and fibrosis^23^,^24^. Decreasing DDR1 expression interrupts this deleterious loop and preserves tissue from further inflammation.

Overexpression of TGF-β1 and of fibrillar collagens were indexes of the development of renal fibrosis^25^ in parallel with the remaining inflammation at the time of sacrifice. Although a direct link between DDR1 activation and TGF-β1 production has not been demonstrated *in vitro*, several *in vivo* studies have observed that genetic deletion of expression of DDR1 is accompanied by downregulation of TGF-β and CTGF in various models of renal disease^2^,^3^,^4^,^5^,^26^,^27^. In the present study, antisense ODNs administration was accompanied by an inhibition of the expression of TGF-β1 and the subsequent activation of Smad3, even when the treatment by antisense was delayed (Fig. 6). The lack of a direct TGFβ response to DDR1 activation in cultured cells *in vitro* can be due to the fact that *in vitro* systems lack the complexity of *in vivo* integrated organs and cell systems. It is also possible that the activation of TGFβ-Smad3 signaling observed *in vivo* is subsequent to the DDR1-mediated inflammatory response. This interaction between DDR1 and TGFβ1-Smad3 signaling can explain, at least partly, the decreased progression of renal fibrosis after the inhibition of DDR1 expression in the antisense groups.

In conclusion, DDR1 appears as an interesting target for therapy of renal disease. Here, we have shown that in severe models of renal disease, pharmaco-genetic inhibition of DDR1 expression by antisense administration can arrest the progression of nephropathy, providing thus for the first time a proof of concept of a treatment based on DDR1 blockade. The development of agents specifically antagonizing the effects of DDR1, such as blocking antibodies and/or DDR1-tyrosine kinase inhibitors^28^,^29^, will offer the possibility to test in experimental models and subsequently in human studies the safety and the efficiency of this approach.

## Methods

### Animal treatment and protocols

All mice were kept in well-controlled animal housing facilities and had free access to water and pellet food. Animal procedures and protocols were in accordance with the European Guidelines for the Care and use of Laboratory Animals and have been approved by the Inserm and UPMC ethical committees.

#### NTS protocol

Glomerulonephritis was induced by retro-orbitaly injection of decomplementated nephrotoxic serum (NTS) prepared as previously described^4^. A total of 90 female mice 129/SV aged 3–6 months and weighting 18–25 g were used (Janvier, Le Genest-St-Isle, France). NTS was injected in 60 mice (23 μl/gBW/day) during three consecutive days. After NTS injections, mice were divided into six groups of 10 mice each. Two groups consisted of mice receiving antisense ODNs directed against DDR1 treatment started 4 or 8 days after NTS (respectively groups As4 and As8). Two groups received scrambled sequences at day 4 or 8 after NTS (groups Scr4 and Scr8) and two received physiological serum at day 4 or 8 (PS4 and PS8).

The remaining 30 mice were divided to 6 groups of 5 animals each receiving antisense or scrambled ODNS or physiological serum (NaCl 0.9%) either at day 4 or at day 8, but without NTS. Since no difference was observed in the values of different parameters of these groups, they were pooled and are presented as one control group in results.

#### UUO protocol

Surgery was performed under general anesthesia (intraperitoneal injection of ketamin 100 mg/kg/Xylazin 20 mg/kg) on 20 129/Sv mice aged 3 month-old (Janvier, Le Genest-St-Isle, France). The left ureter was ligated at two separate points through a left flank incision as previously described^4^. Non-obstructed sham kidneys were used as controls. After the surgery, mice were divided into four groups. Two groups received antisense ODNs directed against DDR1 (UUO + As n = 12, Sham As n = 2), and two other groups received non-specific scrambled ODNs (UUO + Scr n = 8, Sham Scr n = 2). Since Sham As did not differ from Sham Scr, the data of these two groups were pooled. Antisense injections started at day 2 post surgery. All animals were sacrificed at day 7.

A cocktail of 3 specific antisense ODNs was designed as previously described^4^ (Integrated DNA Technologies, Coralville, IA, USA) to block DDR1 expression. Scrambled sequences consisted on same nucleotide composition, but in a random sequence. The sequences used were as follows:

mDDR1 AS 1 Flc: C*A*C*TCCCAAGCCATCCA*C*C*T Flc

mDDR1 AS 6 Flc: C*T*A*TTGCTCCCTCTGTT*C*C*C Flc

mDDR1 AS 8 Flc: G*T*C*CTTCCAGTCCATCC*A*G*C Flc

mDDR1 SCR 1 Flc: A*C*C*CACACACCGACTCC*T*T*C Flc

mDDR1 SCR 6 Flc: C*G*T*CCTCTTACTCGTCC*T*T*C Flc

mDDR1 SCR 8 Flc: C*G*T*CCTCTTACTCGTCC*T*T*C Flc

Antisense and scrambled ODNs were diluted in physiological serum and injected intra peritonealy every 48 h (100 pmol/ODN/injection).

### Proteinuria and BUN

All mice were acclimated in metabolic cages with free access to food and tap water for 24-hour urine collection. Proteinuria, expressed as protein g/mmol of creatininuria, was assessed every 48 h using the Pyrogallol Red method and utilizing a KONELAB automate (Thermo Scientific, Waltman, MA). BUN were assessed in blood plasma obtained on the day of sacrifice. BUN was measured using an enzymatic spectrophotometric method and was expressed as mmol/L.

### Masson’s trichrome staining

Kidneys were fixed in alcohol-formalin-acetic acid, embedded in paraffin, cut into 4-μm sections, and stained with Masson’s trichrome solution. At least twenty photos (ten at ×10 magnification and ten at ×20 magnification) were taken for each mouse, taking care to have a representative view of the total parenchyma. Crescent formation was defined as glomeruli exhibiting two or more layers of cells in Bowman’s space, with or without podocyte injury. The proportion affected of glomeruli was determined by examining photos with ×20 magnification, evaluating a minimum of 50 glomeruli per mouse, and a score based on the percentage of glomeruli presenting crescents was calculated for each mouse. Tubular dilation was evaluated in a scale score form 0–4, and glomerulosclerosis as percentage of sclerotic glomeruli. Crescent-like lesions were defined as glomeruli containing one or two layers of cells in Bowman’s space and/or have filled the Bowman’s space by extra-capillary cells. Histological analysis was performed by two investigators independently.

### Sirius red morphometric analysis

Interstitial fibrosis was analyzed on 5 μm-thick Sirius red-stained paraffin sections at 40× magnification, under polarized light. Interstitial fibrosis was quantified using computer-based morphometric analysis software (Axioplan, Axiophot2, Zeiss, Germany). Measurements were independently performed in a blinded manner on coded slides by two renal pathologists. Data were expressed as the mean value of the percentage of positive area examined.

### Immunohistochemistry and immunofluorescence

Immunohistochemistry was performed on 4 μm thick paraffin embedded sections. Antigens were unmasked with citrate buffer (pH = 6) and sections were treated with peroxidase blocking (Maxvision Bioscience INC) followed by BSA 2%. Samples were stained with primary antibodies: rat anti-F4/80 (AbD Serotec, Paris, France), rabbit anti-CD3ε (DakoFrance, Trappes, France), rabbit anti-DDR1 (Cell signaling, Yvelines, France) and rabbit anti-p-SMAD3 (Abcam, Paris, France) all diluted 1:200 in overnight at 4 °C. AEC (3-Amino-9-Ethylcarbazol, Dako) was used as substrate. Tissues were then stained with hematoxylin QS (Vector, Burlingame, CA) and mounted. Staining of F4/80 and CD3ε cells was quantified using computer-based morphometric analysis software (Axioplan, Axiophot2, Zeiss, Germany). Measurements were independently performed in a blinded manner on coded slides. Data were expressed as the mean value of the percentage of positive area examined. Immunofluorescence was performed on 4 μm thick paraffin sections. Rabbit anti-DDR1 (Cell Signaling, Yvelines, France – diluted 1:200 in overnight at 4 °C) and rabbit anti-CD3ε (DakoFrance, Trappes, France, diluted 1:200 in overnight at 4 °C) antibodies were used as primary. Both secondary antibodies were diluted 1:1000 (Invitrogen Alexa, Saint Aubin, France) and incubated for 1h at room temperature. Images were taken in the same area in the two serial sections.

### Western Blot

Proteins were extracted from total kidney using RIPA lysis buffer supplemented with sodium orthovanadate, PMSF, a protease inhibitor cocktail (Tebu bio, Le Perray en Yvelines, France) and sodium fluorure 10mM. Concentrations were determined using the Bradford assay and 25 μg of protein were run on NuPAGE 4/12% electrophoresis gels (Invitrogen), then transferred on a PVDF membrane (Immobilon-p, Millipore, St Quentin en Yvelines, France). Immunoblotting was performed using rabbit specific primary antibodies anti-DDR1 (Cell signaling, Yvelines, France, diluted 1:1000 in overnight at 4 °C) and anti-GAPDH (Sigma-aldrich, Lyon, France diluted 1:50000 in overnight at 4 °C) for loading control. Then, the membrane was incubated with horseradish peroxidase-linked donkey secondary antibody (GE Healthcare Life Sciences, Saclay, France). The revelation was performed with ECL plus kit (GE Healthcare). Densitometric analysis on Image J was then performed for quantification.

### qRT-PCR on renal tissue

RNA was extracted from renal tissue at the sacrifice using TRI Reagent (Euromedex, Mundolsheim, France). After digestion with DNase I, RNA was reverse transcribed with the Maxima RT Kit (Fermentas). The cDNA obtained was then amplified by PCR in a LightCycler 480 (Roche Diagnostics, Meylan, France) with SYBR Green (Fast Start DNA Master SYBR Green I; Roche Diagnostics) and specific primers for target mRNAs designed using the Universal Probe Library Roche website under the following conditions: 95 °C for 5 min, 45 cycles at 95 °C for 15 s and 60 °C for 15 s, and 72 °C for 15 s. PCR was also carried out for β-actin or Hypoxanthine-guanine phosphoribosyltransferase (HPRT), as housekeeping genes. Results are expressed as 2^−∆Ct^, where Ct is the cycle threshold number normalized to the mean for each corresponding control group. Dissociation curves were analyzed after each run for each amplicon in order to determine the specificity of quantification when using SYBR Green. The sequences (sense and antisense) of the primers used were the following:

β-actin: 5′-AAGAGCTATGAGCTGCCTGA-3′ and 5′-ACGGATGTCAACGTCACACT-3′

HPRT: 5′-GGAGCGGTAGCACCTCCT-3′ and 5′-CTGGTTCATCATCGCTAATCAC-3′

DDR1: 5′-CTCCACCCCATTCTGCAC-3′ and 5′-CAGAAGGAGGCGGTAGGC-3′

TGF-β1: 5′-TGGAGCAACATGTGGAACTC-3′ and 5′-GTCAGCAGCCGGTTACCA-3′

IL-1β: 5′-TGTAATGAAAGACGGCACACC-3′ and 5′-TCTTCTTTGGGTATTGCTTGG-3′

MCP-1: 5′-CATCCACGTGTTGGCTCA-3′ and 5′-GATCATCTTGCTGGTGAATGAGT-3′

Coll1α2: 5′-GCAGGTTCACCTACTCTGTCCT-3′and 5′-CTTGCCCCATTCATTTGTCT-3

Coll3α1: 5′-TCCCCTGGAATCTGTGAATC-3′ and 5′-TGAGTCGAATTGGGGAGAAT3-3

TNF-α: 5′-TCTTCTCATTCCTGCTTGTGG-3′ and 5′-ATGAGAGGGAGGCCATTTG-3′.

### Statistical Analysis

Statistics were realized using the program Logiciel Statview (SAS Institute). Results were expressed as mean ± standard error (SE), and were considered as significant for p < 0.05. For the comparison between groups, ANOVA, Fisher test and Wilcoxon-Mann-Whitney test were used when necessary.

## Additional Information

**How to cite this article**: Kerroch, M. *et al.* Protective effects of genetic inhibition of Discoidin Domain Receptor 1 in experimental renal disease. *Sci. Rep.* **6**, 21262; doi: 10.1038/srep21262 (2016).
